# Association of Early Age at Establishment of Chronic Hepatitis B Infection with Persistent Viral Replication, Liver Cirrhosis and Hepatocellular Carcinoma: A Systematic Review

**DOI:** 10.1371/journal.pone.0069430

**Published:** 2013-07-19

**Authors:** Yusuke Shimakawa, Hong-Jing Yan, Naho Tsuchiya, Christian Bottomley, Andrew J. Hall

**Affiliations:** 1 Faculty of Epidemiology and Population Health, London School of Hygiene and Tropical Medicine, London, United Kingdom; 2 Institutes of Biomedical Sciences, Fudan University, Shanghai, China; Centers for Disease Control and Prevention, United States of America

## Abstract

Age at infection with hepatitis B virus (HBV) is a known risk factor for chronic HBV infection. However, in addition, there is some evidence that early age at infection further increases the risk of primary liver cancer beyond its association with increased risk of chronic infection. This systematic review of observational studies assesses the association between age at initiation of chronic HBV infection and liver cirrhosis, hepatocellular carcinoma, and their predictors including indicators of ongoing viral replication and hepatic damage. The review includes birth order and maternal HBV serology as proxies for age at infection. Electronic searches in two English-language (Medline and Embase, until Jan 2012) and two Chinese-language (CNKI and SinoMed, until Sep 2012) databases without language restriction and manual search through reference lists identified 7,077 papers, of which 19 studies of 21 outcomes (8 primary liver cancer, 1 liver cirrhosis, 10 viral replication and 2 liver inflammation) are included. One study directly examined the age at infection in a longitudinal cohort, 12 assessed maternal sero-status and 6 investigated birth order. The direction of associations in all studies was in accordance with our hypothesis that earlier age at infection is associated with worse outcomes in addition to its effect of increasing the probability of chronic HBV infection. This has implications for the control of hepatitis B.

## Introduction

Chronic infection with the hepatitis B virus (HBV) is associated with an increased risk of liver cirrhosis and hepatocellular carcinoma (HCC) [1]. The prevalence of chronic hepatitis B (CHB) infection varies considerably according to geographical regions, as does the incidence of HCC, and highly endemic areas of HBV infection such as East Asia or sub-Saharan Africa have high HCC incidence [2]. However, the risk of HCC amongst people with CHB infection also varies markedly by geographical areas. Population-based cohort studies in hepatitis B surface antigen (HBsAg)-positive men have estimated age-standardized HCC incidence rates of 451–1,030/100,000 carrier-years in East Asia [3], [4], 68/100,000 carrier-years in sub-Saharan Africa [5] and 34/100,000 carrier-years in the Mediterranean basin [6]. This variation in incidence appears to be due to variation in HBV natural history as is discussed below.

Persistence of high HBV viral load [7] or e antigenemia [8] play an important role in increasing the risk of primary liver cancer, and variations in this may explain the observed geographical difference in HCC incidence. In Taiwan, about half of HBsAg-positive children remain hepatitis B e antigen (HBeAg)-positive into their twenties [9], while in sub-Saharan Africa where HBV endemicity is similar, the prevalence of HBeAg among HBsAg-positive people declines to 10% in the second decade of life [10]. Evans *et al*. attempted to identify factors responsible for the persistence of high viral replication in an ecological study comparing Chinese and African populations. They suggested that earlier age at HBV infection and the subsequent prolonged maintenance of viral replication in Asians with CHB infection is the cause of the higher HCC incidence in Asia [5]. Indeed, there is wide geographical variation in the mode of HBV transmission which may explain the differences in the average age at which persistent infection is established. In sub-Saharan Africa or the Mediterranean basin, horizontal transmission during childhood is the major mode [11]. In contrast, perinatal transmission from the mother is important in Asia where 40% of chronic infections were attributable to this mode in the pre-vaccination era [12].

Age at infection is known to influence the establishment of HBV infection. Infection persists in 80–90%, 20–30%, <10%, and <5% of people infected perinatally, in early childhood, adolescence, and adulthood, respectively [13]. However, it is unclear whether early HBV infection also increases the risk of liver cirrhosis and/or HCC through the persistence of high viral replication, in addition to increasing the risk of chronic infection [14]. We therefore undertook a systematic review of observational studies where the association between age at establishment of chronic HBV infection and risk of cirrhosis and/or HCC has been investigated among people chronically infected with HBV.

In addition to liver cirrhosis and HCC as endpoints, two indicators of ongoing viral replication (serum HBV DNA level, presence or persistence of viral replication (HBeAg)) and two of hepatic damage (elevated serum alanine transaminase (ALT) and degree of hepatic fibrosis) were included. These are important predictive factors for cirrhosis and HCC, and often used as indicators for antiviral treatment of CHB infection [15]. Our hypothesis is that earlier age at infection is associated with worse outcomes in addition to its effect of increasing the probability of chronic HBV infection.

## Materials and Methods

### Inclusion Criteria

#### Types of participants

CHB infection was defined as serum HBsAg positivity on two occasions at least 6 months apart. However, because new HBV infections in adults are uncommon in highly endemic areas where the vast majority of HBsAg-positive people acquire the infection perinatally or during childhood, HBsAg positivity on only one occasion in adults living in highly prevalent communities was assumed to reflect chronic carriage of HBsAg [5].

#### Exposures of interest

The age at the time of infection with HBV was estimated by one of the following: 1) direct measurement through frequent serological examination of an uninfected cohort to determine the time point at which a person sero-converted from negative- to positive-HBsAg; 2) the HBV serological profile (HBsAg and/or HBeAg) of the mother of the participant; or 3) the person’s birth order. We expect early age at infection to be negatively associated with birth order in an endemic area, because, in the absence of immunization, early-born children are exposed to common infectious pathogens after they start to go to the school, whereas later-born children are often exposed much earlier through their older siblings [16]. In contrast, we expect early age at infection with HBV to be positively associated with having a HBsAg-positive (and especially HBeAg-positive) mother, because a chronically infected individual with seropositive mother has a higher likelihood to have been infected through perinatal transmission than an individual with CHB infection who was born to a seronegative mother. Maternal history of HBV infection was not considered in the review because it is frequently unknown, and absence of a history does not necessarily indicate that the mother is negative for HBV marker.

#### Outcomes of interest

Quantitative/qualitative serum HBV DNA, presence of serum HBeAg, levels of serum ALT, degree of liver fibrosis, cirrhosis, and HCC.

#### Types of studies

Any observational studies (i.e., cross-sectional, case-control or cohort) published in any language which met all of the following criteria were included: the study examined the association between any of the exposures of interest and any of the outcomes of interest described above; individuals without CHB infection were excluded from the analyses (because the primary focus of this review is the link between age at infection and the risk of HCC beyond its effect of increasing risk of CHB infection); in studies of maternal HBV sero-status, the subjects with unknown maternal sero-status were excluded from the analyses to avoid misclassification of exposure status; case series, i.e. studies without a control group, were included if the Greenwood-Yule method, or a related approach, was used to examine the birth order distribution [17].

### Search Strategy

We conducted a systematic search using two English-language databases (Medline, 1946 to Jan 2012 and Embase, 1974 to Jan 2012) and two Chinese-language databases (CNKI, 1979 to Sep 2012 and SinoMed, 1979 to Sep 2012). Subject headings used in Medline search included “hepatitis B”, “alanine transaminase”, “hepatitis B e antigens”, “DNA, viral”, “virus replication”, “liver cirrhosis”, “hepatocellular carcinoma”, “age factors”, “infectious disease transmission, vertical”, and “birth order”. Manual search through reference lists was also conducted. Gray literature was not searched. The full search strategy is reported in Appendix S1.

### Study Selection

Eligibility criteria were specified in advance and documented in a protocol (Appendix S2). The title and abstract of all papers identified by the electronic searches in English databases were screened by two independent reviewers (YS and NT). Those identified in Chinese databases were screened by another reviewer (HY). Papers detected through the screening process were retrieved and reviewed to assess their eligibility. Disagreements were resolved by discussion with a fourth author (AH). Twenty-seven authors of the articles were contacted for clarification of the study design or results. Seventeen responded and nine provided numerical data that were not presented in the published papers. Data extraction was independently carried out by two reviewers for articles from the English databases and by one reviewer for those from the Chinese databases. A standardized pre-piloted data extraction sheet was used (Appendix S3). The included studies were evaluated for the risk of bias (Appendix S4). The aim of assessing the risk of bias was to summarize limitations identified in each study, rather than to exclude additional studies on the basis of low methodological quality.

### Statistical Analysis

For the cross-sectional and case-control studies, odds ratios (OR) were estimated. In longitudinal studies, the risk ratio, rather than the rate ratio, was computed because person years at risk were not available for many of the published papers. The chi-squared test (or Fisher’s exact test for small samples) was used to test the statistical significance of the crude associations. The chi-squared test for trend was presented for the association between birth order and liver disease. For studies where no events were reported in one of the comparison groups, odds ratios were estimated by adding 0.5 to each cell of the contingency table. The Greenwood-Yule method was used to estimate the expected distribution of birth order among cases in studies without a control group, and a statistical test introduced by Haldane and Smith that compares the sum of the observed birth ranks with the sum of expected birth ranks was applied [18]. We present adjusted effect estimates for studies where these were reported in the original analysis. A meta-analysis was not performed because of differences in outcomes and exposures between studies. Potential for publication bias was visually assessed using funnel plots and statistically with Egger’s test [19]. This review was reported in accordance with checklists presented in the PRISMA guidelines (Checklist S1) [20]. All analyses were conducted using STATA 11.0 (Stata Corporation, College Station, Texas).

## Results

**Figure 1:**
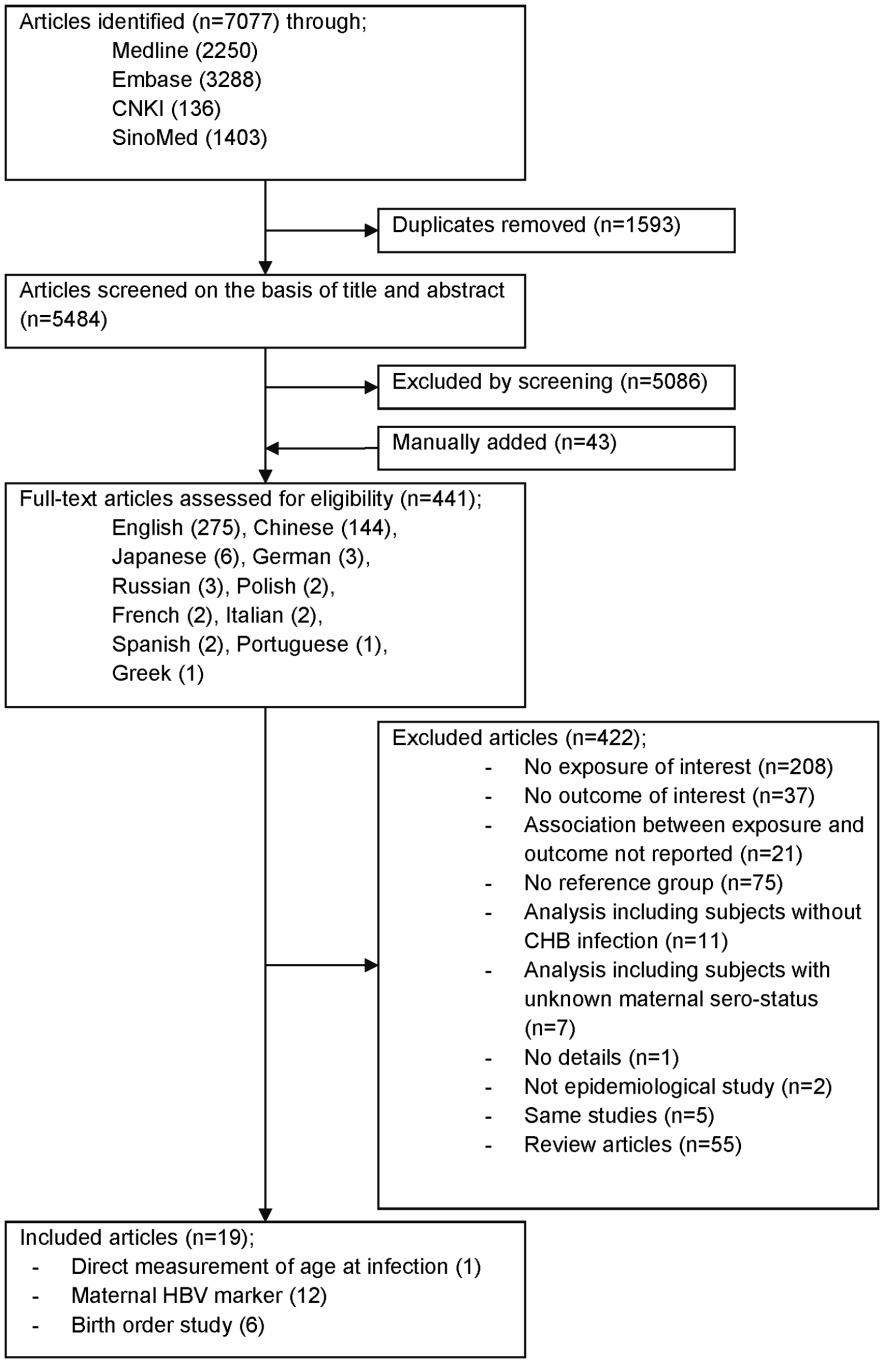
Flow diagram of study selection. Abbreviations: CHB, chronic hepatitis B; HBV, hepatitis B virus.

The search of databases identified 7,077 potential articles (2,250 in Medline, 3,288 in Embase, 136 in CNKI and 1,403 in SinoMed) of which 1,593 were excluded due to duplication. Review of the titles and abstracts excluded 5,086. Forty-three papers were manually identified from reference lists. The full text of the 441 articles was examined in detail, and of these 422 was discarded leaving 19 papers eligible for the systematic review. Criteria of exclusion are described in Figure 1.

There was only one longitudinal study that examined the association between age at first HBsAg-positive result and persistence of HBeAg [21]. This study used an historical cohort of HBsAg-negative children in Alaska who were later identified to have a CHB infection through semi-annual serological follow-up. The cohort was followed up for greater than ten years to assess the timing of HBeAg loss (Table 1).

**Table 1:** Study of the Association Between Time of HBV Infection and the Risk of HBeAg Persistence in a Systematic Review, up to 2012.

First Author, Year (Reference No.)	Region	Study Design	Serological Course	No. of Subjects	Median Age at1^st^ HBsAg-positiveResult	MedianDurationof Follow-up	Median No. of SerologicalTests
McMahon, 2001 [21]	Alaska, USA	Cohortstudy	Remained HBeAg-positive throughout the follow-up period	9	4.6 years old	11.1 years	12
			Lost HBeAg during the course without reversion	47	7.8 years old	16.6 years	17
			Lost HBeAg during the course with reversion to positive HBeAg	11	12.5 years old	14.8 years	20

A total of 12 studies assessed the association of maternal HBV sero-status with various outcomes (Table 2 and 3); HCC (2 case-control studies) [22], [23], cirrhosis by liver histopathology (1 cross-sectional study) [24], persistence of HBeAg (4 cohort studies) [25]–[28], and presence of HBeAg at one time point (5 cross-sectional studies) [29]–[33]. The two cohort studies that evaluated HBeAg loss also examined ALT levels [25], [28]. Except for four studies [26], [30]–[32], the studies exclusively enrolled children. In all the cohort studies, treatment was not given during follow-up except one study of steroid withdrawal therapy [26]. All studies examined HBsAg as the only maternal HBV marker, and three also considered maternal HBeAg [28], [29], [33]. The proportion of participants with available maternal sero-status varied markedly between studies. In two studies having a mother alive [30] or maternal serology available [25] was one of the eligibility criteria while among the remaining studies the percentage of subjects with known maternal sero-status varied from 49% [32] to 95% [27], [28]. The timing of when maternal sero-status was defined in relation to the child’s age also varied. One study in England [29] assessed prenatal maternal HBeAg whereas 6 studies examined mothers’ HBV markers when their children were enrolled in the study (Table 2 and 3). In the remaining 5 studies, the timing was unclear.

**Table 2:** Studies Assessing the Effect of Maternal HBV Sero-status on HCC, Liver Cirrhosis and Peak ALT Levels in a Systematic Review, up to 2012.

						Crude			Adjusted		
First Author, Year, Region (Reference No.)	Study Design	Age Range, Years^a^	Type of Maternal HBV Sero-marker	Frequency in Casesor Exposed Group^b^	Frequency in Controls or Non-exposed Group^c^	OR orRR^d^	95% CI	*P* value	OR or RR	95% CI	*P* value
1. HCC											
Beasley, 1982, Taiwan [22] e	CC	Children	HBsAg^f^	12/14 (86%)	17/49 (35%)	11.3	1.9, 67.8	0.0008	N/R		
Chang, 1989, Taiwan [23]	CC	3–16	HBsAg at entry	29/31 (94%)	22/44 (50%)	14.5^g^	2.5, 84.0	0.0001	N/R		
2. Cirrhosis											
Hsu, 1988, Taiwan [24]	CS	Children	HBsAg^f^	6/23 (23%)	0/21 (0%)	16.0^h^	0.8, 303.6	0.02^i^	N/R		
3. Peak ALT level											
Kojima, 1985, Japan [25]	Co	2–12	HBsAg^f^	3/9 (33%)	22/28 (79%)	0.42	0.17, 1.09	0.04	N/R		
Tseng, 2011, Taiwan [28]	Co	0–16	HBsAg at entry	63/137 (46%)	29/48 (60%)	0.76	0.57, 1.02	0.09	N/R		
			HBeAg at entry	33/80 (41%)	59/105 (56%)	0.73	0.54, 1.00	0.04	N/R		

Abbreviations: ALT, alanine transaminase; CC, case-control study; CI, confidence interval; Co, cohort study; CS, cross-sectional study; HBeAg, hepatitis B e antigen; HBsAg, hepatitis B surface antigen; HCC, hepatocellular carcinoma; HR, hazard ratio; N/R, not reported; OR, odds ratio; RR, risk ratio.

a When age range was not available, study was categorized as children (<20 years old), adults (≥20 years old) or both children and adults.

b Prevalence of exposure in cases presented in case-control design and prevalence of outcome in exposed group presented in cross-sectional and cohort study.

c Prevalence of exposure in controls presented in case-control design and prevalence of outcome in non-exposed group presented in cross-sectional and cohort study.

d Odds ratios for case-control or cross-sectional studies and risk ratios for cohort studies are presented.

e Communication with the author confirmed that all the HCC cases were positive for HBsAg.

f When the measurement of maternal sero-status was performed is not known.

g Matched design without matched analysis.

h As a contingency table contains a zero cell, 0.5 was added to each cell.

i Although the 95% CI does not exceed the unity, *P* value is <0.05 due to the small sample size.

**Table 3:** Studies Assessing the Effect of Maternal HBV Sero-status on Persistence/Prevalence of HBeAg in a Systematic Review, up to 2012.

						Crude			Adjusted		
First Author, Year, Region (Reference No.)	Study Design	Age Range, Years^a^	Type of MaternalHBV Sero-marker	Frequency in Exposed Group	Frequency in Non-exposed Group	OR orRR^b^	95% CI	*P* value	OR orHR^c^	95% CI	*P* value
1. Persistence of HBeAg											
Kojima, 1985, Japan [25]	Co	2–12	HBsAg at entry	7/9 (78%)	9/28 (32%)	2.4	1.3, 4.6	0.02	N/R		
Kojima, 1985, Japan [26]	Co	19–48	HBsAg at entry	4/4 (100%)	2/9 (22%)	4.5	1.3, 15.3	0.02	N/R		
Chang, 1989, Taiwan [27]	Co	0–15	HBsAg^d^	121/142 (85%)	52/75 (69%)	1.2	1.0, 1.5	0.006	N/R		
Tseng, 2011, Taiwan [28]	Co	0–16	HBsAg at entry	56/137 (41%)	8/48 (17%)	2.5	1.3, 4.8	0.002	1.2^e^	0.8, 1.8	0.5
			HBeAg at entry	41/80 (51%)	23/105 (22%)	2.3	1.5, 3.6	<0.0001	1.8^e^	1.1, 2.8	0.01
2. Prevalence of HBeAg											
Wheeley, 1989, UK [29]	CS	0–16	Prenatal HBeAg	32/42 (76%)	0/1 (0%)	9.3^f^	0.4, 245.6	0.1	N/R		
Habu, 1991, Japan [30]	CS	Children & adults	HBsAg at entry	71/101 (70%)	96/152 (63%)	1.4	0.8, 2.4	0.2	1.6^g^	0.9, 2.9	0.1
Tai, 1999, Taiwan [31]	CS	>15	HBsAg at entry	67/221 (30%)	35/131 (27%)	1.2	0.7, 1.9	0.5	N/R		
Hopkirk, 2000, New Zealand [32] h	CS	Children & adults	HBsAg^d^	160/281 (57%)	214/530 (40%)	2.0	1.5, 2.6	<0.0001	1.8^g^	1.3, 2.4	0.0005
Soderstrom, 2002, Sweden [33]	CS	2–18	HBeAg^d^	15/16 (94%)	11/17 (65%)	8.2	0.8, 400.8	0.09	N/R		

Abbreviations: ALT, alanine transaminase; CC, case-control study; CI, confidence interval; Co, cohort study; CS, cross-sectional study; HBeAg, hepatitis B e antigen; HBsAg, hepatitis B surface antigen; HCC, hepatocellular carcinoma; HR, hazard ratio; N/R, not reported; OR, odds ratio; RR, risk ratio.

a When age range was not available, study was categorized as children (<20 years old), adults (≥20 years old) or both children and adults.

b Odds ratios for case-control or cross-sectional studies and risk ratios for cohort studies are presented.

c Except for the study of Tseng et al. [28] which presented hazard ratios, odds ratios are presented.

d When the measurement of maternal sero-status was performed is not known.

e Multivariable model included maternal HBsAg, maternal HBeAg, peak ALT and HBV genotype.

f As a contingency table contains a zero cell, 0.5 was added to each cell.

g Adjusted for age.

h The author provided the raw data to compute odds ratio.

For birth order, one cross-sectional study [34] and two case-control studies [16], [35] compared the frequency distribution of birth order by the presence of HBeAg-positivity and HCC, respectively (Table 4). Three Chinese case series of HBV-related HCC used the Greenwood-Yule method (Table 5) [36]–[38]. All birth order studies were conducted in adults.

**Table 4:** Studies of Birth Order With Control Group in a Systematic Review, up to 2012.

								Crude			Adjusted		
First Author, Year (Reference No.)	Region	Study Design	Age Range, Years^a^	Outcome	BirthOrder	No. ofCases (%)	No. ofControls (%)	OR	95% CI	*P* for trend	OR	95% CI	*P* for trend
**Hsieh, 1992** [16]	Greece	CC	Adults	HCC	1^st^	17 (20%)	9 (27%)	1.0	Reference	0.04	1.0^b^	Reference	0.02
					2^nd^	11 (13%)	11 (33%)	0.5	0.2, 1.7		0.9	0.2, 4.2	
					3^rd^	17 (20%)	3 (9%)	3.0	0.7, 13.8		7.8	1.4, 42.4	
					4^th^	15 (18%)	4 (12%)	2.0	0.5, 8.0		4.0	0.8, 20.7	
					≥5^th^	25 (29%)	6 (18%)	2.2	0.6, 7.6		3.8	0.9, 16.5	
**Kuper, 2000** [35]	Greece	CC	Adults	HCC	1^st^	42 (20%)	13 (43%)	1.0	Reference	0.0008	1^st^ 1.0^c^	Reference	N/R
					2^nd^	59 (28%)	9 (30%)	2.0	0.8, 5.2		2^nd^ 2.0	0.8, 5.3	
					3^rd^	49 (24%)	7 (23%)	2.2	0.8, 6.0		≥3^rd^ 4.1	1.3, 12.7	
					≥4^th^	58 (28%)	1 (4%)	18.0	2.0, 163.4				
**Tai, 2002** [34]	Taiwan	CS	Adults	HBeAg	1^st^	29 (29%)	65 (23%)	1.00	Reference	0.09	N/R^d^		
					2^nd^	29 (29%)	68 (25%)	0.96	0.51, 1.77				
					3^rd^	24 (24%)	78 (28%)	0.69	0.36, 1.30				
					4^th^	11 (11%)	40 (14%)	0.62	0.28, 1.38				
					≥5^th^	7 (7%)	27 (10%)	0.58	0.23, 1.50				

Abbreviations: CC, case-control study; CI, confidence interval; CS, cross-sectional study; HBeAg, hepatitis B e antigen; HCC, hepatocellular carcinoma; N/R, not reported; OR, odds ratio.

a When age range was not available, study was categorized as children (<20 years old), adults (≥20 years old) or both children and adults.

b Adjusted for age, sex, smoking and anti-HCV.

c Adjusted for age, sex, smoking, alcohol, schooling, anti-HCV and sibship size.

d Stratification by relationship with index case (i.e., children and siblings) was reported.

**Table 5:** Studies of Birth Order Without Control Group in a Systematic Review, up to 2012.

First Author, Year (Reference No.)	Region	StudyDesign	Age Range, Years^a^	Outcome	Birth Order	Observed Distribution	Expected Distribution	Greenwood-Yule Method	Haldane-Smith Method
Cai, 2003 [36]	Haimen, China	Case series	>36	HCC	1^st^	29	26.27	1.10	Observed<Expected, t = 2.17, df = 121, *P* = 0.03
					2^nd^	28	22.27	1.26	
					3^rd^	18	17.27	1.04	
					4^th^	9	12.94	0.70	
					≥5^th^	10	15.30	0.65	
Cao, 2005 [37]	Luoyang, China	Case series	Adults	HCC	1^st^	19	16.75	1.13	Observed<Expected, t = 0.95, df = 62, *P* = 0.4
					2^nd^	15	13.75	1.09	
					3^rd^	11	12.25	0.90	
					4^th^	10	9.25	1.08	
					≥5^th^	8	11.00	0.73	
Song, 2009 [38]	Shunde, China	Case series	Adults	HCC	1^st^	17	13.92	1.22	Observed<Expected, t = 2.20, df = 46, *P* = 0.03
					2^nd^	15	10.92	1.37	
					3^rd^	5	7.42	0.67	
					4^th^	4	5.75	0.70	
					≥5^th^	6	8.98	0.67	

Abbreviations: df, degree of freedom; HCC, hepatocellular carcinoma.

a When age range was not available, study was categorized as children (<20 years old), adults (≥20 years old) or both children and adults.

a Ratio of observed/expected number is presented.

Except for one study [28], all longitudinal studies compared proportions rather than incidence rates. Results of multivariable analyses were only reported in three studies [16], [28], [35] although some studies presented results stratified by age group [27], [30]. The author of a New Zealand study [32] provided additional data which enabled us to control for age. The risk of bias in the included studies is summarized in Appendix S5.

### Time of HBV Infection Study

In the Alaskan study children who remained HBeAg-positive were infected younger (median 4.6 years) than children who lost HBeAg with subsequent reversion to HBeAg positive (median 12.5 years) or without reversion (median 7.8 years, Table 1).

### Maternal HBV Serology Studies

**Figure 2:**
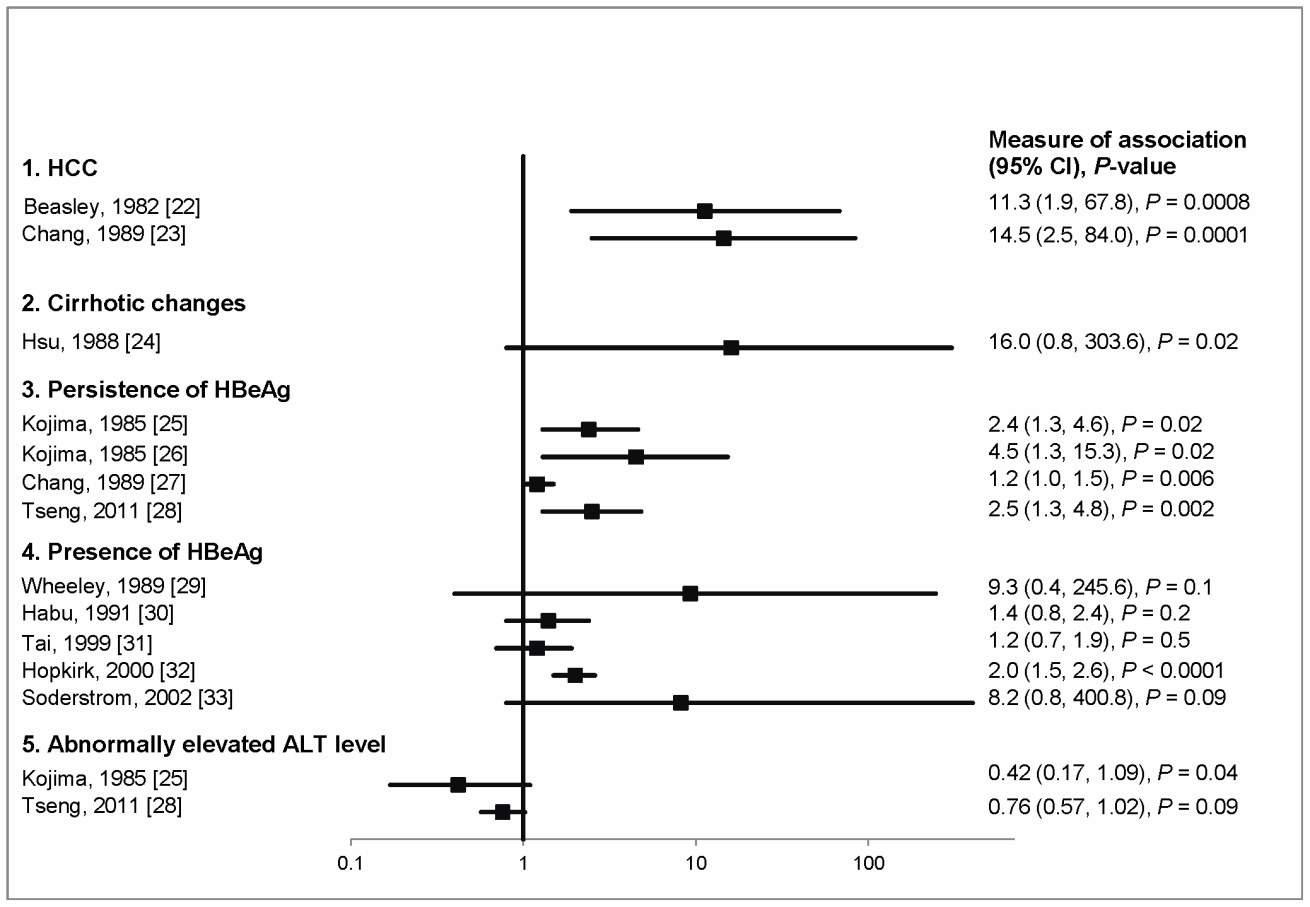
Effect measures and 95% CIs for the association between maternal HBV sero-status and HBV-related outcomes. Odds ratios for case-control or cross-sectional studies [22]–[24], [29]–[33] and risk ratios for cohort studies [25]–[28] are presented. Except the study by Wheeley [29] which assessed maternal HBeAg, all studies examined maternal HBsAg.

Three pediatric studies from Taiwan, two with HCC and one with cirrhotic changes in liver histopathology as outcome, revealed a similar magnitude of association with having an infected mother (ORs range from 11.3 to 16.0, Table 2 and Figure 2). One longitudinal study of treatment-induced HBeAg loss in adults and three studies of spontaneous HBeAg loss in children found good evidence for an association of HBeAg persistence with having an HBsAg-positive mother (Table 3 and Figure 2). However, in one of these [28] the association was no longer statistically significant after adjusting for maternal HBeAg, peak ALT and HBV genotype (hazard ratio 1.2, 95% confidence interval (CI): 0.8, 1.8). Another study [27] stratified the analysis by age at study entry and observed heterogeneity (test of homogeneity *P*<0.0001) in the risk ratios; 0.87 (95% CI: 0.79, 0.95), 1.1 (0.8, 1.4) and 1.3 (1.0, 1.7) in age group of <1, 1–6 and >6 years old, respectively. Tseng *et al.* [28] evaluated maternal HBeAg in addition to maternal HBsAg and found a good evidence for the association with children’s HBeAg persistence in univariable analysis (risk ratio 2.3, 95% CI: 1.5, 3.6) and after controlling for maternal HBsAg, peak ALT and HBV genotype (hazard ratio 1.8, 95% CI: 1.1, 2.8).

Of five cross-sectional studies of HBeAg prevalence (Table 3 and Figure 2), one from New Zealand [32] showed strong evidence for an association with positive maternal HBsAg and this remained after controlling for age (OR 1.8, 95% CI: 1.3, 2.4). The pediatric study of immigrants in Sweden found weak evidence (OR 8.2, 95% CI: 0.8, 400.8, *P* = 0.09) for an association with having an HBsAg-positive mother [33]. Although the other studies did not demonstrate a statistically significant difference in HBeAg prevalence between those born to seropositive and seronegative mothers, the direction of the association was positive. Habu *et al*. [30] found no evidence of an association after adjusting for age (OR 1.6, 95% CI: 0.9, 2.9).

Two longitudinal cohort studies of children positive for HBeAg assessed peak ALT levels during follow-up (Table 2 and Figure 2) [25], [28]. In both studies, there was good evidence that the proportion that experienced abnormally high ALT levels was smaller in children born to HBsAg-positive mothers than in those born to negative mothers.

### Birth Order Studies With a Control Group

Both Greek studies found that later birth order is associated with a higher risk of HCC (Table 4). This association did not change after adjusting for other prognostic factors. A Taiwanese cross-sectional study of HBeAg prevalence showed weak evidence for the association between earlier birth order and higher risk of positive HBeAg.

### Birth Order Studies without Control Group

A study from Haimen, China demonstrated that there was a higher than expected frequency of HCC patients in birth orders 1–3, and a lower than expected frequency in birth orders higher than 4 (Table 5). The Haldane-Smith method confirmed statistical evidence (*P* = 0.03) of this. A study from Shunde district was consistent with these findings. And the association was in the same direction but not statistically significant in a study from Luoyang (*P* = 0.4).

### Publication Bias

**Figure 3:**
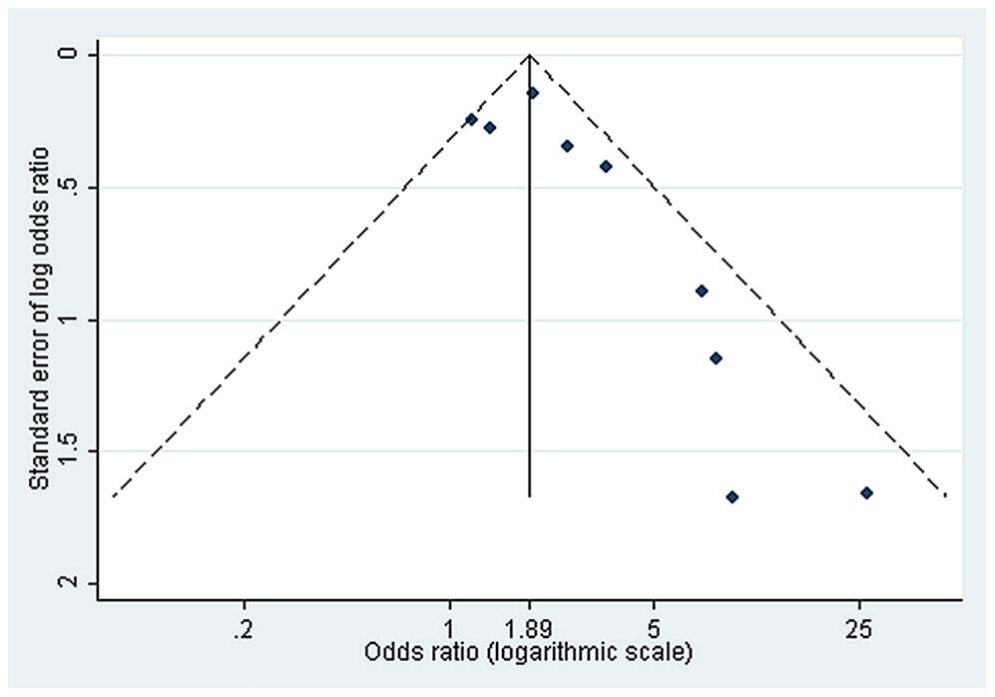
Funnel plot for studies investigating the relation between maternal sero-marker and the persistence/presence of HBeAg. Dashed line represents pseudo 95% confidence limits.

A funnel plot is presented for the studies examining the association between the maternal HBV marker and the persistence/prevalence of HBeAg (Figure 3). The plot appears asymmetric (which is indicative of publication bias) and this is confirmed by Egger’s test (P = 0.04). There were too few studies to assess publication bias for other associations.

## Discussion

Of 21 outcomes examined in 19 studies, univariable analysis of 14 outcomes supported our hypothesis with *P*≤0.05 and two with weak evidence (0.05<*P*<0.1). Although the other studies did not reach statistical significance, the direction of the associations was consistent with our hypothesis.

McMahon *et al.* directly assessed the age at HBV infection and its association with HBeAg persistence in Alaskan natives whose approximate date of infection was known because of consecutive serological tests. They showed that the median age at first HBsAg-positive result was lower in those who did not clear HBeAg during the follow-up period than those who HBeAg sero-converted, suggesting that the early age at infection is associated with delayed HBeAg loss. However, the length of follow up is different for the two groups, which is a crucial determinant of the chance of HBeAg loss.

Studies of maternal HBV serology showed that having a mother with a positive HBV marker is associated with worse outcomes (HCC, cirrhotic changes, persistence of HBeAg or possession of HBeAg at one particular time) although three of these were not supported by statistical evidence. Two longitudinal studies from East Asia which evaluated both HBeAg loss and peak ALT levels during follow-up demonstrated good evidence that children with HBsAg-positive mothers experienced elevated levels of hepatic enzymes less frequently and fewer episodes of HBeAg loss than those whose mothers are negative. It is well established that having a highly elevated serum ALT level during the immune-tolerant phase of CHB infection is a factor leading to early HBeAg seroconversion [39].

The only maternal serological study which reported the results of a multivariable analysis [28] showed that the association with maternal HBsAg observed in the univariable analysis was no longer significant after controlling for maternal HBeAg. This reflects the fact that maternal HBeAg is a stronger predictor of perinatal transmission than maternal HBsAg. In fact, the risk of perinatal transmission ranges from 10–20% in HBsAg-positive mothers without HBeAg to 90% in mothers with positive HBeAg [40]. Nevertheless, because HBeAg sero-clearance (loss of the maker) occurs faster than HBsAg sero-clearance in chronically infected persons (2–15% versus 1% per year) [15], misclassification of maternal HBeAg status between when the index child was born and when he/she entered the study is greater than misclassification of maternal HBsAg status. Apart from 5 studies not reporting when mothers were bled, all but one examined maternal sero-status at the child’s study entry, which might have led to non-differential misclassification of the exposure, resulting in underestimation of its association. The only study which investigated prenatal maternal HBeAg [29], the best proxy for perinatal transmission, was inconclusive because of a small sample size.

Recent evidence suggests that viral genotypes modify the natural history of CHB infection. Certain genotypes are associated with delayed HBeAg seroconversion [41] and also HCC risk [42]. Moreover, associations between certain viral genotypes and mother-to-infant transmission have been reported [43]. For example, Tseng *et al.* [28] found that genotype C was associated with delayed HBeAg seroconversion after accounting for maternal HBsAg and HBeAg status. The study also showed that maternal HBeAg was associated with persistence of HBeAg after adjusting for viral genotype.

Two Greek case-control studies of birth order demonstrated that HCC cases are concentrated in higher birth orders, suggesting that later-born children who might have been exposed to HBV at a young age through their older siblings have a higher risk of developing HCC than early-born children. However this finding was not confirmed in three subsequent Chinese birth order studies which applied the Greenwood-Yule methods. Furthermore, one Taiwanese study showed that HBeAg was more prevalent in earlier birth orders although the evidence was weak [34]. These heterogeneities can be understood in terms of geographic differences in the main mode of transmission. In East Asia, perinatal infection through vertical transmission is relatively important compared to other parts of the world [12]. In such places, first-born children have a higher chance of having been born to a HBeAg-positive mother than later-born children, because young mothers with positive-HBeAg will clear it with increasing age [15]. Consequently, early-born children have a higher chance of having been infected earlier from their mother than later-born children. In contrast, in Mediterranean countries where children are horizontally infected through other children with positive-HBeAg [11], birth order acts as an indicator for number of older siblings, and later-born children have an increased chance of being exposed to HBV at an early age through infectious older siblings than early-born children. Perinatal transmission also occurs in Greece but is much less common than in China. This could explain the results of the first Greek study where people born as the second child have lower odds of HCC than the first birth rank (OR 0.5), with the odds increased for higher birth orders.

The funnel plot and Egger’s test suggest the presence of publication bias in the studies assessing the association between maternal sero-marker and persistence/presence of HBeAg. In general, observational studies are more vulnerable to publication bias than randomized trials. Epidemiological studies are often conducted retrospectively by analysing existing databases, and unless published, this kind of study is hard to trace. In addition, often only statistically significant variables are reported [19]. Although we were unable to assess the publication bias in the birth order studies due to the small number of studies included, these studies are prone to selective reporting bias because birth order is easily obtained by interview without additional cost.

Although a robust conclusion cannot be drawn due to the potential role of publication bias and the heterogeneity of studies included in the review, this systematic review supports the hypothesis that earlier age at HBV infection is associated with an increased risk of HCC through persistence of viral replication. This review has highlighted some issues in interpreting this association. Studies using maternal HBsAg are only informative when prenatal maternal HBeAg status is available in the study population. The relationships between surrogate markers of disease endpoints, such as viral load and/or HBeAg, are now so clear that they can be used to shorten the time period over which the association is observed. Finally, confounding factors such as viral genotype should be appropriately controlled in the analyses.

The implications of the effect of early age at infection on the prevention of cirrhosis and primary liver cancer are twofold. First it means that the impact of hepatitis B vaccination on raising the average age at infection will not simply be in reducing the prevalence of chronic infection but also in reducing the adverse effects of that chronic infection in those acquiring it at an older age. Second it adds emphasis to the critical importance of interrupting perinatal transmission – as reflected in the WHO recommendation for a birth dose within 24 hours of birth in all countries [44].

## Supporting Information

Appendix S1 **Search strategy.**
(PDF)

Appendix S2 **Protocol for the systematic review.**
(PDF)

Appendix S3 **Data extraction sheet.**
(XLSX)

Appendix S4 **A framework for assessing the risk of bias in individual studies.**
(PDF)

Appendix S5 **Description of risk of bias.**
(PDF)

Checklist S1 **PRISMA 2009 checklist**
(DOC)

## References

[1] IARC Working Group on the Evaluation of Carcinogenic Risks to Humans (2011) A review of human carcinogens: biological agents. Lyon, France.

[2] Lavanchy D (2004) Hepatitis B virus epidemiology, disease burden, treatment, arid current and emerging prevention and control measures. J Viral Hepat 11: 97–107. 14996343 10.1046/j.1365-2893.2003.00487.x

[3] Yang HI, Lu SN, Liaw YF, You SL, Sun CA, et al (2002) Hepatitis B e antigen and the risk of hepatocellular carcinoma. N Engl J Med 347: 168–174. 12124405 10.1056/NEJMoa013215

[4] Evans AA, Chen G, Ross EA, Shen FM, Lin WY, et al (2002) Eight-year follow-up of the 90,000-person Haimen City cohort: I. Hepatocellular carcinoma mortality, risk factors, and gender differences. Cancer Epidemiol Biomarkers Prev 11: 369–376. 11927497

[5] Evans A, Connell APO, Pugh JC, Mason S (1998) Geographic variation in viral load among hepatitis B carriers with differing risks of hepatocellular carcinoma. Cancer Epidemiol Biomarkers Prev 7: 559–565. 9681522

[6] Ribes J, Clèries R, Rubió A, Hernández JM, Mazzara R, et al (2006) Cofactors associated with liver disease mortality in an HBsAg-positive Mediterranean cohort: 20 years of follow-up. Int J Cancer 119: 687–694 doi:10.1002/ijc.21882 16496403 10.1002/ijc.21882

[7] Chen CJ, Yang HI, Su J, Jen CL, You SL, et al (2006) Risk of hepatocellular carcinoma across a biological gradient of serum hepatitis B virus DNA level. JAMA 295: 65–73 doi:10.1001/jama.295.1.65 16391218 10.1001/jama.295.1.65

[8] Chen YC, Chu CM, Liaw YF (2010) Age-specific prognosis following spontaneous hepatitis B e antigen seroconversion in chronic hepatitis B. Hepatology. 51: 435–444. 10.1002/hep.23348 19918971

[9] Chu CM, Liaw YF (2007) Chronic hepatitis B virus infection acquired in childhood: special emphasis on prognostic and therapeutic implication of delayed HBeAg seroconversion. J Viral Hepat 14: 147–152 doi:10.1111/j.1365-2893.2006.00810.x 17305879 10.1111/j.1365-2893.2006.00810.x

[10] Mendy ME, McConkey SJ, van der Sande MAB, Crozier S, Kaye S, et al (2008) Changes in viral load and HBsAg and HBeAg status with age in HBV chronic carriers in The Gambia. Virol J 5: 49 doi:10.1186/1743-422X-5-49 18416832 10.1186/1743-422X-5-49 PMC2358882

[11] Hadziyannis SJ (2011) Natural history of chronic hepatitis B in Euro-Mediterranean and African countries. J Hepatol 55: 183–191 doi:10.1016/j.jhep.2010.12.030 21238520 10.1016/j.jhep.2010.12.030

[12] Edmunds WJ, Medley GF, Nokes DJ, O’Callaghan CJ, Whittle HC, et al (1996) Epidemiological patterns of hepatitis B virus (HBV) in highly endemic areas. Epidemiol Infect 117: 313–325. 8870629 10.1017/s0950268800001497 PMC2271713

[13] Edmunds WJ, Medley GF, Nokes DJ, Hall AJ, Whittle HC (1993) The influence of age on the development of the hepatitis B carrier state. Proc Biol Sci 253: 197–201 doi:10.1098/rspb.1993.0102 8397416 10.1098/rspb.1993.0102

[14] Taylor BC, Yuan J-M, Shamliyan T a, Shaukat A, Kane RL, et al (2009) Clinical outcomes in adults with chronic hepatitis B in association with patient and viral characteristics: A systematic review of evidence. Hepatology 49: 85S–95S doi:10.1002/hep.22929 10.1002/hep.22929 19399797

[15] Liaw YF, Chu CM (2009) Hepatitis B virus infection. Lancet 373: 582–592 doi:10.1016/S0140-6736(09)60207-5 19217993 10.1016/S0140-6736(09)60207-5

[16] Hsieh CC, Tzonou A, Zavitsanos X, Kaklamani E, Lan SJ, et al (1992) Age at first establishment of chronic hepatitis B virus infection and hepatocellular carcinoma risk: A birth order study. Am J Epidemiol 136: 1115–1121. 1334366 10.1093/oxfordjournals.aje.a116577

[17] Greenwood M, Yule GU (1914) On the Determination of Size of Family and of the Distribution of Characters in Order of Birth from Samples Taken Through Members of the Sibships. J R Stat Soc 77: 179–199.

[18] Haldane J, Smith CAB (1947) A Simple exact test for birth-order effect. Ann Eugenics 14: 117–124. 10.1111/j.1469-1809.1947.tb02383.x 18863975

[19] Egger M, Smith GD, Altman D (2001) Systematic Reviews in Health Care: Meta-analysis in Context. Wiley-Blackwell.

[20] Liberati A, Altman DG, Tetzlaff J, Mulrow C, Gøtzsche PC, et al (2009) The PRISMA statement for reporting systematic reviews and meta-analyses of studies that evaluate health care interventions: explanation and elaboration. J Clin Epidemiol 62: e1–34 doi:10.1016/j.jclinepi.2009.06.006 19631507 10.1016/j.jclinepi.2009.06.006

[21] McMahon BJ, Holck P, Bulkow L, Snowball M (2001) Serologic and clinical outcomes of 1536 Alaska Natives chronically infected with hepatitis B virus. Ann Intern Med 135: 759–768. 11694101 10.7326/0003-4819-135-9-200111060-00006

[22] Beasley RP (1982) Hepatitis B virus as the etiologic agent in hepatocellular carcinoma - epidemiologic considerations. Hepatology 2: 21S–26S.

[23] Chang MH, Chen DS, Hsu HC, Hsu HY, Lee CY (1989) Maternal transmission of hepatitis B virus in childhood hepatocellular carcinoma. Cancer 64: 2377–2380. 2553245 10.1002/1097-0142(19891201)64:11<2377::aid-cncr2820641130>3.0.co;2-8

[24] Hsu HC, Lin YH, Chang MH, Su IJ, Chen DS (1988) Pathology of chronic hepatitis B virus infection in children: with special reference to the intrahepatic expression of hepatitis B virus antigens. Hepatology 8: 378–382. 3356420 10.1002/hep.1840080232

[25] Kojima M, Yasuda M, Tanaka H, Adachi N (1985) Natural seroconversion of HBe antigen to anti-HBe in HBs antigen carrier children-From the difference of the modes of HBV transmission [Japanese]. Acta Hepatologica Japonica 26: 1139–1145.

[26] Kojima M, Shimizu M, Adachi N, Takahashi Y, Tsuda F (1985) Factors predicting effects of withdrawal of steroid therapy for chronic hepatitis B. Carrier state of mothers of patients [Japanese]. Acta Hepatologica Japonica 26: 796.

[27] Chang MH, Sung JL, Lee CY, Chen CJ, Chen JS, et al (1989) Factors affecting clearance of hepatitis B e antigen in hepatitis B surface antigen carrier children. Journal of Pediatrics 115: 385–390. 2769497 10.1016/s0022-3476(89)80836-4

[28] Tseng YR, Wu JF, Ni YH, Chen HL, Chen CC, et al (2011) Long-term effect of maternal HBeAg on delayed HBeAg seroconversion in offspring with chronic hepatitis B infection. Liver Int 31: 1373–1380. 21745315 10.1111/j.1478-3231.2011.02574.x

[29] Wheeley SM, Tarlow MJ, Boxall EH (1989) Chronic hepatitis B in male and female children of HBsAg carrier mothers. J Hepatol 8: 226–231. 2715622 10.1016/0168-8278(89)90011-1

[30] Habu D, Monna T, Saitoh S, Kuroki T, Kobayashi K (1991) Relationship between the condition of the liver in patients and carriers with hepatitis B virus (HBV) and whether there is intrafamilial clustering of HBV [Japanese]. Nihon Shokakibyo Gakkai Zasshi 88: 1545–1553. 1942609

[31] Tai DI, Changchien CS, Hung CS, Chen CJ (1999) Replication of hepatitis B virus in first-degree relatives of patients with hepatocellular carcinoma. Am J Trop Med Hyg 61: 716–719. 10586900 10.4269/ajtmh.1999.61.716

[32] Hopkirk N, Moyes CD, Lucas CR (2000) Liver function and hepatitis markers in carriers of hepatitis B virus in New Zealand. N Z Med J 113: 114–116. 10834277

[33] Soderstrom A, Norkrans G, Conradi N, Krantz M, Horal P, et al (2002) Histologic activity of childhood chronic hepatitis B related to viremia levels, genotypes, mutations, and epidemiologic factors. J Pediatr Gastroenterol Nutr 35: 487–494. 12394372 10.1097/00005176-200210000-00006

[34] Tai DI, Lo SK, Kuo CH, Du JM, Chen CJ, et al (2002) Replication of hepatitis B in HBsAg-positive siblings. J Viral Hepat 9: 272–279. 12081604 10.1046/j.1365-2893.2002.00353.x

[35] Kuper H, Hsieh C, Stuver SO, Mucci LA, Tzonou A, et al (2000) Birth order, as a proxy for age at infection, in the etiology of hepatocellular carcinoma. Epidemiology 11: 680–683. 11055629 10.1097/00001648-200011000-00011

[36] Cai R, Meng W, Lu H, Jiang F (2003) A study on the relationship of birth order hepatocellular carcinoma [Chinese]. Zhonghua Liu Xing Bing Xue Za Zhi 24: 22–25. 12678957

[37] Cao Y, Bian J, Guo H (2005) Birth Order Analysis of Primary Liver Cancer in Luoyang [Chinese]. Fudan University Journal of Medical Science 32: 447–450.

[38] Song SF, Chen SD, Gao YH (2009) Birth order and primary liver cancer in Shunde, Guangdong [Chinese]. Chinese Journal of Public Health 25: 1326–1327.

[39] Liaw YF (2003) Hepatitis flares and hepatitis B e antigen seroconversion: Implication in anti-hepatitis B virus therapy. J Gastroenterol Hepatol 18: 246–252 doi:10.1046/j.1440-1746.2003.02976.x 12603523 10.1046/j.1440-1746.2003.02976.x

[40] Okada K, Kamiyama I, Inomata M, Imai M, Miyakawa Y, et al (1976) E Antigen and Anti-E in the Serum of Asymptomatic Carrier Mothers as Indicators of Positive and Negative Transmission of Hepatitis B Virus to Their Infants. N Engl J Med 294: 746–749 doi:10.1056/NEJM197604012941402 943694 10.1056/NEJM197604012941402

[41] Livingston SE, Simonetti JP, Bulkow LR, Homan CE, Snowball MM, et al (2007) Clearance of Hepatitis B e Antigen in Patients With Chronic Hepatitis B and Genotypes A, B, C, D, and F. Gastroenterology. 133: 1452–1457. 10.1053/j.gastro.2007.08.010 17920063

[42] Kew MC, Kramvis A, Yu MC, Arakawa K, Hodkinson J (2005) Increased hepatocarcinogenic potential of hepatitis B virus genotype A in Bantu-speaking sub-saharan Africans. J Med Virol 75: 513–521 doi:10.1002/jmv.20311 15714494 10.1002/jmv.20311

[43] Wen WH, Chen HL, Ni YH, Hsu HY, Kao JH, et al (2011) Secular trend of the viral genotype distribution in children with chronic hepatitis B virus infection after universal infant immunization. Hepatology 53: 429–436. 21274864 10.1002/hep.24061

[44] WHO (2010) Hepatitis B vaccines: WHO position paper–recommendations. Vaccine 28: 589–590 doi:10.1016/j.vaccine.2009.10.110 19896455 10.1016/j.vaccine.2009.10.110

