# Supplementary material for: Association of Early Age at Establishment of Chronic Hepatitis B Infection with Persistent Viral Replication, Liver Cirrhosis and Hepatocellular Carcinoma: A Systematic Review
Source: PLoS One. 2013 Jul 19;8(7):e69430. doi: 10.1371/journal.pone.0069430 (PMC3716646; doi:10.1371/journal.pone.0069430)
Supplement: Appendix S1 — Search strategy. (PDF) [file pone.0069430.s001.pdf]

Appendix S1. Search Strategy

(1) Electronic Searches

Following electronic databases were searched using the OvidSP search platform:

- MEDLINE (1946 to January (week 2) 2012);
- EMBASE (1974 to January (week 2) 2012);
- CNKI (1979 to September (week 2) 2012);
- SinoMed (1979 to September (week 2) 2012).

(1-1) MEDLINE

#1 explode “Hepatitis B”/ all subheadings or explode “Hepatitis B virus”/ all subheadings

#2 hepatitis b or hepatitis-b or (type b adj1 hepatitis) or hbv or hep b or hbsag or hbs-ag or hbs antigen* or hbs-antigen* or hepatitis b surface antigen* or hepatitis-b surface antigen*

#3 “Alanine Transaminase”/ all subheadings

#4 alanine transaminase* or alanine aminotransferase* or glutamic pyruvate transaminase* or ALT or ALAT or GPT or SGPT

#5 “Hepatitis B e Antigens”/ all subheadings

#6 hbeag or hbe-ag or hbe antigen* or hbe-antigen* or (hepatitis b adj3 e antigen*) or (hepatitis-b adj3 e antigen*) or ((type b adj1 hepatitis) adj3 e antigen*) or (hbv adj3 e antigen*)

#7 “DNA, viral”/ all subheadings or “Virus replication”/ all subheadings

#8 dna or replication* or viral titre or viral titer or viral load

#9 “Liver Cirrhosis”/ all subheadings

#10 cirrho* or ((liver or hepat*) adj3 fibro*)

#11 “Carcinoma, Hepatocellular”/ all subheadings

#12 ((liver or hepat*) adj3 (cancer* or carcinom* or neoplasm* or malign* or tumo?r*)) or hcc or hepatoma

#13 “Age Factors”/ all subheadings

#14 age adj5 infect*

#15 “Infectious Disease Transmission, Vertical”/ all subheadings

#16 perinatal or peri-natal or vertical or MTCT or mother-to-child or mother to child or mother-child or mother child or mother-to-infant or mother to infant or mother-infant or mother infant or maternal-to-child or maternal to child or maternal-child or maternal child or maternal-to-infant or maternal to infant or maternal-infant or maternal infant or adult-to-child or adult to child or adult-child or adult child or adult-to-infant or adult to infant or adult-infant or adult infant or maternal-f?etal or maternal f?etal or f?etomaternal

#17 postnatal or post-natal or horizontal or child-to-child or child to child or child-child or child child or sibling-to-sibling or sibling to sibling or

sibling-sibling or sibling sibling or between children or between siblings or between family or within household

#18 “Birth Order”/ all subheadings

#19 birth adj3 (order* or link*)

#20 (serolog* or seropositiv* or serostatus) adj5 (famil* or parent* or father* or paternal or mother* or maternal or sibling* or brother* or sister*)

#21 (hepatitis b or hepatitis-b or (type b adj1 hepatitis) or hbv or hep b or hbsag or hbs-ag or hbs antigen* or hbs-antigen* or hepatitis b surface antigen* or hepatitis-b surface antigen*) adj5 (famil* or parent* or father* or paternal or mother* or maternal or sibling* or brother* or sister*)

#22 #1 or #2

#23 #3 or #4 or #5 or #6 or #7 or #8 or #9 or #10 or #11 or #12

#24 #13 or #14

#25 #15 or #16 or #17

#26 #18 or #19

#27 #20 or #21

#28 #22 and #23 and #24

#29 #22 and #23 and #25

#30 #22 and #23 and #26

#31 #22 and #23 and #27

#32 #28 or #29 or #30 or #31

(1-2) EMBASE

#1 “hepatitis B”/ all subheadings or “hepatitis b B virus”/ all subheadings

#2 hepatitis b or hepatitis-b or (type b adj1 hepatitis) or hbv or hep b or hbsag or hbs-ag or hbs antigen* or hbs-antigen* or hepatitis b surface antigen* or hepatitis-b surface antigen*

#3 “alanine aminotransferase”/ all subheadings

#4 alanine transaminase* or alanine aminotransferase* or glutamic pyruvate transaminase* or ALT or ALAT or GPT or SGPT

#5 “hepatitis B(e) antigen”/ all subheadings

#6 hbeag or hbe-ag or hbe antigen* or hbe-antigen* or (hepatitis b adj3 e antigen*) or (hepatitis-b adj3 e antigen*) or ((type b adj1 hepatitis) adj3 e antigen*) or (hbv adj3 e antigen*)

#7 “virus DNA”/ all subheadings or “virus replication”/ all subheadings

#8 dna or replication* or viral titre or viral titer or viral load

#9 “liver cirrhosis”/ or “decompensated liver cirrhosis”/ all subheadings

#10 cirrho* or ((liver or hepat*) adj3 fibro*)

#11 “liver cancer”/ or “ascites hepatoma”/ or “liver carcinoma”/ or “liver cell carcinoma”/ all subheadings

#12 (liver or hepat*) adj3 (cancer* or carcinom* or neoplasm* or malign* or tumo?r*) or hcc or hepatoma

#13 “age”/ all subheadings

#14 age adj5 infect*

#15 “vertical transmission”/ all subheadings

#16 perinatal or peri-natal or vertical or MTCT or mother-to-child or mother to child or mother-child or mother child or mother-to-infant or mother to infant or mother-infant or mother infant or maternal-to-child or maternal to child or maternal-child or maternal child or maternal-to-infant or maternal to infant or maternal-infant or maternal infant or adult-to-child or adult to child or adult-child or adult child or adult-to-infant or adult to infant or adult-infant or adult infant or maternal-f?etal or maternal f?etal or f?etomaternal

#17 postnatal or post-natal or horizontal or child-to-child or child to child or child-child or child child or sibling-to-sibling or sibling to sibling or sibling-sibling or sibling sibling or between children or between siblings or between family or within household

#18 “birth order”/ all subheadings

#19 birth adj3 (order* or link*)

#20 (serolog* or seropositiv* or serostatus) adj5 (famil* or parent* or father* or paternal or mother* or maternal or sibling* or brother* or sister*)

#21 (hepatitis b or hepatitis-b or (type b adj1 hepatitis) or hbv or hep b or hbsag or hbs-ag or hbs antigen* or hbs-antigen* or hepatitis b surface antigen* or

hepatitis-b surface antigen*) adj5 (famil* or parent* or father* or paternal or mother* or maternal or sibling* or brother* or sister*)

#22 #1 or #2

#23 #3 or #4 or #5 or #6 or #7 or #8 or #9 or #10 or #11 or #12

#24 #13 or #14

#25 #15 or #16 or #17

#26 #18 or #19

#27 #20 or #21

#28 #22 and #23 and #24

#29 #22 and #23 and #25

#30 #22 and #23 and #26

#31 #22 and #23 and #27

#32 #28 or #29 or #30 or #31

(1-3) CNKI

(关键词=乙肝 or 乙型肝炎 or HBV or HBsAgor 乙肝表面抗原) AND (关键词=ALT or 丙氨酸氨基转移酶 or HBeAg or 乙肝核心抗原 or 肝癌 or 肝细胞癌 or 肝硬化 or 肝纤维化 or HBVDNAor HBV-DNA) AND (关键词=年龄 or 出生顺序分析 or 垂直传播 or 水平传播 or 家庭)

(1-4) SinoMed

1.主题词:肝炎，乙型，慢性/全部树/全部副主题词 -限定:-

2.全部字段:乙肝 -限定:-

3.全部字段:乙型肝炎 -限定:-

4.全部字段:HBV -限定:-

5.全部字段:hbsag -限定:-

6.主题词:丙氨酸转氨酶/全部树/全部副主题词 -限定:-

7.全部字段:ALT -限定:-

8.全部字段:丙氨酸氨基转移酶 -限定:-

9.主题词:肝炎 e 抗原，乙型/全部树/全部副主题词 -限定:-

10.全部字段:HBeAg -限定:-

11.全部字段:乙肝 e 抗原 -限定:-

12.主题词:病毒复制/全部树/全部副主题词 -限定:-

13.全部字段:DNA -限定:-

14.全部字段:复制 -限定:-

15.全部字段:病毒滴度 -限定:-

16.全部字段:病毒载量 -限定:-

17.主题词:肝硬化/全部树/全部副主题词 -限定:-

18.全部字段:肝纤维化 -限定:-

19.主题词:肝肿瘤/全部树/全部副主题词 -限定:-

20.主题词:年龄因素/全部树/全部副主题词 -限定:-

21.主题词:疾病传播，垂直/全部树/全部副主题词 -限定:-

22.全部字段:母婴传播 -限定:-

23.主题词:疾病传播，水平/全部树/全部副主题词 -限定:-

24.全部字段:水平传播 -限定:-

25.主题词:出生顺序/全部树/全部副主题词 -限定:-

26.全部字段:出生顺序分析 -限定:-

27.主题词:家庭/全部树/全部副主题词 -限定:-

28.#5 or #4 or #3 or #2 or #1 -限定:-

29.#19 or #18 or #17 or #16 or #15 or #14 or #13 or #12 or #11 or #10 or #9 or #8 or #7 or #6 -限定:-

30.#24 or #23 or #22 or #21 -限定:-

31.#29 and #28 and #20 -限定:-

32.#26 or #25 -限定:-

33.#30 and #29 and #28 -限定:-

34.#32 and #29 and #28 -限定:-

35.#29 and #28 and #27 -限定:-

36.#35 or #34 or #33 or #31 -限定:-

(2) Additional Searches

Additional studies were sought manually by checking reference lists of all included papers. None of the searches were restricted by date, language or publication status.
