# Supplementary material for: Association of Early Age at Establishment of Chronic Hepatitis B Infection with Persistent Viral Replication, Liver Cirrhosis and Hepatocellular Carcinoma: A Systematic Review
Source: PLoS One. 2013 Jul 19;8(7):e69430. doi: 10.1371/journal.pone.0069430 (PMC3716646; doi:10.1371/journal.pone.0069430)
Supplement: Appendix S2 — Protocol for the systematic review. (PDF) [file pone.0069430.s002.pdf]

Appendix S2. Protocol for the systematic review

Effect of Age at HBV Infection on the Risk of High HBV DNA Level, Persistent Viral Replication, Elevation of Serum Alanine Transaminase Level, Advanced Liver Firbrosis and HCC in Individuals with CHB Infection: A Systematic Review

1. Objectives

To review the association between the age at HBV infection and risk of high HBV DNA level, persistent viral replication, elevation of serum alanine transaminase level, advanced fribrosis and/or HCC in persons with chronic hepatitis B infection

2. Criteria for Considering Studies for this Review

(1) Types of Studies

Any studies whether interventional or observational, reported in any language, irrespective of publication status. Studies must have a reference group to make a comparison. The only exception is a case series using the Greenwood-Yule method or its related approaches to examine the effect of birth order, because these methods compare the observed birth order distribution of affected individuals with the expected distribution and do not require control group.[1] In a case-control design, a control group which only consists of people chronically infected with HBV must be reported.

(2) Types of Participants

Participants of any age who were found to have CHB infection at some stage of study are considered. Chronic hepatitis B is defined as a condition proven by serum HBsAg positivity on two occasions at least 6 months apart. However, because new HBV infections in adults are not so common in highly endemic areas where the vast majority of HBsAg positive people acquire the infection perinatally or during childhood, HBsAg positivity on only one occasion in an adult living in highly prevalent communities is assumed to reflect chronic carriage of HBsAg.[2]

(3) Exposure of interest

The age at the time of infection with HBV is estimated either by:

- Direct measurement through frequent follow-up examination of an uninfected cohort to determine the time point at which a person seroconverted to positive HBsAg
- HBV serological profile of the mother of the participant
- Person’s birth order

(4) Outcome of Interest

- Levels of serum alanine transaminase (ALT)
- Presence of serum hepatitis B e antigen (HBeAg)

- Quantitative/qualitative serum HBV DNA
- Liver fibrosis determined by either liver histology or non-invasive tests
- Cirrhosis
- HCC

3. Data Collection

(1) Study Selection

First, the title and abstract of all papers identified by the electronic searches will be screened by two independent reviewers, by applying the inclusion criteria. Second, papers detected through screening process will be retrieved and reviewed to assess the eligibility. When there is any doubt whether a paper can be included, clarification will be sought from the author of the paper. Disagreements will be resolved by discussion with a third author. Only studies which fulfilled all inclusion criteria will be included in the review.

(2) Data Extraction

Data extraction will be carried out, by using a standardised pre-piloted data extraction sheet. The information included in the sheet is summarised in Appendix 1.

4. Assessment of Risk of Bias

The included studies will be evaluated for the risk of bias by modified framework that was introduced by Altman[3] (Appendix 2).

5. Data Analysis

For the study of maternal HBV serological profile, the odds ratio (OR) of having a worse outcome (high HBV DNA load (>2,000 IU/ml), positive HBeAg, elevated serum ALT (>40 U/L), advanced liver fibrosis (Metavir F≥2, or equivalent value in non-invasive tests), cirrhosis or HCC) in participants who have seropositive mother compared with those with seronegative mother will be calculated. For the birth order study, the odds of outcomes in each rank of birth will be compared with the odds in the first-born child as the reference. Measure of effect, its 95% confidence interval and p-value will be all reconstructed from the information reported in each included article. The results of multivariable analyses reported in the original paper will be presented without any modification in this review. STATA version 11 will be used for all analyses. This protocol was made in accordance with checklists presented in the PRISMA guideline.[4]

6. References

1. MacMahon B, Thomas F P, Johannes I. *Epidemiologic methods*. Little, Brown; 1960:302.

2. Evans A, Connell APO, Pugh JC, Mason S. Geographic variation in viral load among hepatitis B carriers with differing risks of hepatocellular carcinoma. *Cancer Epidemiol Biomarkers Prev*. 1998;7:559–565.

3. Egger M, Smith GD, Altman D. *Systematic Reviews in Health Care: Meta-analysis in Context*. Wiley-Blackwell; 2001:512.

4. Liberati A, Altman DG, Tetzlaff J, et al. The PRISMA statement for reporting systematic reviews and meta-analyses of studies that evaluate health care interventions: explanation and elaboration. *J Clin Epidemiol*. 2009;62(10):e1–34.

Appendix 1. Information Included in the Standardised Data Extraction Sheet

- First author
- Country or region of study
- Year of study
- Study design
- Selection of participants
- Characteristics of participants studied (age, sex, whether asymptomatic, whether cirrhotic, prevalence of HBsAg, definition of cases and controls in case-control study, participation rate, drop-out rate in cohort study)
- Exposures of interest:
    - If age at infection was directly measured, the interval between each follow-up, test used for detect seroconversion
    - If serological profile of participant's mother was assessed, what serological test was performed and when the sample was taken (before the birth of study participant or current serostatus)
    - If birth order was examined, how the information was collected and the definition that the authors used
- Outcomes of interest:
    - Definition of high HBV DNA level, which method was used to measure this
    - Method used to measure HBeAg
    - Definition of elevated ALT level, which method was used to measure this
    - Definition of advanced fibrosis, which criteria was used when liver histology was performed, and which method was used when non-invasive test was carried out
    - Case definition of cirrhosis
    - Case definition of HCC
- Follow-up of participants (in a cohort study)
- Univariable analyses
- Multivariable analyses and confounding factors adjusted for

Appendix 2. A Framework for Assessing the Risk of Bias in Individual Studies

Adapted from the framework presented by Altman[3]

Study feature	Qualities sought				
1. Sample of patients	Eligibility criteria defined	Good	Poor	N/R	N/A
	Sample selection explained (setting, locations and periods of recruitment)	Good	Poor	N/R	N/A
	Representative (unbiased selection of controls)	Good	Poor	N/R	N/A
2. Outcome	Fully defined	Good	Poor	N/R	N/A
	Known for all or a high proportion of patients	>80%	60-79%	<60%	N/R
	Outcome assessor blinded to exposure status	Good	Poor	N/R	N/A
3. Prognostic variable	Fully defined, including details of method of sampling	Good	Poor	N/R	N/A
	Available for all or a high proportion of patients	>80%	60-79%	<60%	N/R
	Exposure assessor blinded to outcome status	Good	Poor	N/R	N/A
4. Analysis	Appropriate control for confounding factors	Good	Poor	N/R	N/A
	Appropriate statistical method	Good	Poor	N/R	N/A

Abbreviations; N/R, not reported: and N/A, not applicable.
