# Supplementary material for: Association of Early Age at Establishment of Chronic Hepatitis B Infection with Persistent Viral Replication, Liver Cirrhosis and Hepatocellular Carcinoma: A Systematic Review
Source: PLoS One. 2013 Jul 19;8(7):e69430. doi: 10.1371/journal.pone.0069430 (PMC3716646; doi:10.1371/journal.pone.0069430)
Supplement: Appendix S4 — A framework for assessing the risk of bias in individual studies. (PDF) [file pone.0069430.s004.pdf]

Appendix S4. A Framework for Assessing the Risk of Bias in Individual Studies

Adapted from the framework presented by Altman (Egger M, Smith GD, Altman D, eds. *Systematic Reviews in Health Care: Meta-analysis in Context.* Wiley-Blackwell; 2001).

Study feature	Qualities sought				
1. Sample of patients	Eligibility criteria defined	Good	Poor	N/R	N/A
	Sample selection explained (setting, locations and periods of recruitment)	Good	Poor	N/R	N/A
	Clinical and demographic characteristics fully described	Good	Poor	N/R	N/A
	Representative (unbiased selection of controls)	Good	Poor	N/R	N/A
	Assembled at a common stage in the course of their disease	Good	Poor	N/R	N/A
	Completeness	Good	Poor	N/R	N/A
2. Follow	Sufficiently long	Good	Poor	N/R	N/A

up of patients					
3. Outcome	Fully defined	Good	Poor	N/R	N/A
	Known for all or a high proportion of patients	>80%	60-79%	<60%	N/R
	Outcome assessor blinded to exposure status	Good	Poor	N/R	N/A
4. Prognostic variable	Fully defined, including details of method of measurement (maternal HBsAg study: when maternal serology was investigated?)	Good	Poor	N/R	N/A
	Available for all or a high proportion of patients	>80%	60-79%	<60%	N/R
	Exposure assessor blinded to outcome status	Good	Poor	N/R	N/A
5. Analysis	Continuous predictor variable analysed appropriately	Good	Poor	N/R	N/A
	Appropriate control for	Good	Poor	N/R	N/A

	confounding factors				
	Appropriate statistical method	Good	Poor	N/R	N/A
6. Treatment subsequent to inclusion in cohort	Fully described	Good	Poor	N/R	N/A
	Treatment standardized or randomised	Good	Poor	N/R	N/A

Abbreviations: N/A, not applicable; N/R, not reported.
