# Supplementary material for: Association of Early Age at Establishment of Chronic Hepatitis B Infection with Persistent Viral Replication, Liver Cirrhosis and Hepatocellular Carcinoma: A Systematic Review
Source: PLoS One. 2013 Jul 19;8(7):e69430. doi: 10.1371/journal.pone.0069430 (PMC3716646; doi:10.1371/journal.pone.0069430)
Supplement: Appendix S5 — Description of risk of bias. (PDF) [file pone.0069430.s005.pdf]

Appendix S5-1. Description of Risk of Bias in Case-Control Studies, Cross-Sectional Studies and Case Series

First Author, Year, (Reference No.)	1. Sample of Patients			2. Outcome			3. Exposure			4. Analysis	
	Eligibility Criteria	Sample Selection	Representativeness in Controls	Fully Defined	Blinded to Exposure Status	Known for All Subjects?	Fully Defined	Blinded to Outcome Status	Known for All Subjects?	Confounding Factors Adjusted for	Appropriate Analysis
Beasley, 1982 [22]	N/R	N/R	N/R	N/R	N/R	N/R	N/R	N/R	N/R	none	good
Chang , 1989 [23]	good	poor[b]	poor[d]	poor[e]	N/A	100%	good	N/R	65%	matched on age	poor[e]
Hsu, 1988 [24]	poor[a]	poor[c]	N/A	good	N/R	100%	poor[g]	N/R	67%	none	good
Wheeley, 1989 [29]	poor[a]	poor[c]	N/A	good	N/R	100%	good	N/A	90%	none	poor[m]
Habu, 1991	poor[a]	poor[c]	N/A	good	N/R	100%	poor[h]	N/R	100%[k]	stratified by age group	good

[30]											
Tai, 1999 [31]	good	good	N/A	good	N/R	90%	poor[i]	N/R	52%	none	poor[m]
Hopkirk, 2000 [32]	N/R	poor[c]	N/A	poor[h]	N/R	89%	poor[g]	N/R	49%	adjusted for age	good
Soderstrom, 2002 [33]	good	poor[c]	N/A	good	N/R	100%	poor[g]	N/R	75%	none	good
Hsieh, 1992 [16]	good	good	good	good	good	100%	good	N/R	100%	adjusted for age, sex, smoking, anti-HCV, HBsAg and sibship size	poor[n]
Kuper, 2000 [35]	good	poor[b]	good	poor[e]	N/A	100%	good	N/R	99% for cases and 97% for controls	matched on age and sex, adjusted for age, sex, schooling, smoking, alcohol, anti-HCV, HBsAg and sibship size	poor[l, n]

Tai, 2002 [34]	good	good	N/A	good	N/R	95%	good	N/R	100%	stratified by relationship with index case	good
Cai, 2003 [36]	good	poor[c]	N/A	good	N/R	100%	poor[j]	N/A	100%	none	poor[o]
Cao, 2005 [37]	good	poor[c]	N/A	poor[f]	N/R	100%	poor[j]	N/A	100%	none	poor[o]
Song, 2009 [38]	good	good	N/A	good	N/R	96%	poor[j]	N/A	100%	none	poor[o]

Abbreviations: HBsAg, hepatitis B surface antigen; HCV, hepatitis C virus; N/A, not applicable; N/R, not reported.

[a] Definition of HBsAg carrier was not presented.

[b] The way of selecting control subjects was unclear.

[c] Setting of sample selection was unclear.

[d] Hospital-based cases were compared with population-based controls.

[e] Cut-off value of alpha fetoprotein (AFP) for hepatocellular carcinoma (HCC) diagnosis was not presented.

[f] Diagnostic criteria for HCC were not presented.

[g] When maternal sero-status was examined was unclear.

[h] Method of testing hepatitis B virus (HBV) marker was not presented.

[i] When maternal sero-status was examined was not presented in the original paper, however this was described in a subsequent paper of the same study [34].

[j] Birth order was not defined.

[k] Maternal sero-status was known in all the participants because having a mother alive was one of the eligibility criteria.

[l] Matched design, but no matched analysis.

[m] Clustering effect of being born to the same mother was not taken account.

[n] Two different control groups were combined.

[o] Both the Greenwood-Yule and Haldane-Smith method are prone to bias due to change in population dynamics.

Appendix S5-2. Description of Risk of Bias in Cohort Studies

First Author, Year, (Reference No.)	1. Sample of Patients			2. Follow up	3. Outcome			4. Exposure			5. Analysis		6. Treatment After Inclusion in Cohort
	**Eligibility Criteria**	**Sample Selection**	**Assembled at a Common Stage**	**Sufficiently Long**	**Fully Defined**	**Blinded to Exposure Status**	**Known for all Subjects?**	**Fully Defined**	**Blinded to Outcome Status**	**Known for all Patients?**	**Confounding Factors Adjusted for**	**Appropriate Analysis**	**Fully Described**
McMahon, 2001 [21]	good	poor[b]	Good	good	good	N/R	100%	poor[e]	N/A	100%	N/R	poor[i]	poor[j]
Kojima, 1985 [25]	poor[a]	poor[b]	Good	good	poor[c]	N/R	100%	poor[f]	N/R	100%[h]	N/R	poor[i]	good (no treatment)

Kojima, 1985 [26]	poor[a]	poor[b]	Good	good	poor[d]	N/R	100%	poor[f]	N/R	72%	N/R	poor[i]	good (steroid)
Chang, 1989 [27]	good	poor[b]	Good	good	good	N/R	100%	poor[g]	N/R	95%	Stratified by age group	poor[i]	good (no treatment)
Tseng, 2011 [28]	good	poor[b]	Good	good	good	N/R	100%	good	N/R	95%	adjusted for HBV genotype and maternal HBV marker	good	good (no treatment)

Abbreviations: HBV, hepatitis B virus; N/A, not applicable; N/R, not reported.

[a] Definition of hepatitis B surface antigen (HBsAg) carrier was not presented.

[b] Setting of sample selection was unclear.

[c] Method of measuring serum alanine transaminase (ALT) was not presented.

[d] Method of testing HBV marker was not presented.

[e] How often blood sample was obtained to determine timing of HBV infection was not reported.

[f] When maternal sero-status was examined was not presented in this paper, but this was confirmed by the communication with the author.

[g] When maternal sero-status was examined was unclear.

[h] Maternal sero-status was known in all the participants because having a mother tested was one of the eligibility criteria.

[i] Person-years at risk were not taken account in the analysis.

[j] For the sub-study of patients with known age at HBV infection, number of those who were treated was not reported.
